# Quantitative prediction of oral cancer risk in patients with oral leukoplakia

**DOI:** 10.18632/oncotarget.17550

**Published:** 2017-05-02

**Authors:** Yao Liu, Yicheng Li, Yue Fu, Tong Liu, Xiaoyong Liu, Xinyan Zhang, Jie Fu, Xiaobing Guan, Tong Chen, Xiaoxin Chen, Zheng Sun

**Affiliations:** ^1^ Department of Oral Medicine, Beijing Stomatological Hospital, Capital Medical University, Beijing, China; ^2^ Cancer Research Program, Julius L. Chambers Biomedical Biotechnology Research Institute, North Carolina Central University, Durham, North Carolina, USA; ^3^ Department of Pathology, Beijing Stomatological Hospital, Capital Medical University, Beijing, China; ^4^ Beijing Institute of Dental Research, School of Stomatology, Capital Medical University, Beijing, China; ^5^ Division of Medical Oncology, Department of Internal Medicine, The Ohio State University, Columbus, Ohio, USA

**Keywords:** exfoliative cytology, oral cancer risk, oral leukoplakia, oral squamous cell carcinoma, quantitative prediction

## Abstract

Exfoliative cytology has been widely used for early diagnosis of oral squamous cell carcinoma. We have developed an oral cancer risk index using DNA index value to quantitatively assess cancer risk in patients with oral leukoplakia, but with limited success. In order to improve the performance of the risk index, we collected exfoliative cytology, histopathology, and clinical follow-up data from two independent cohorts of normal, leukoplakia and cancer subjects (training set and validation set). Peaks were defined on the basis of first derivatives with positives, and modern machine learning techniques were utilized to build statistical prediction models on the reconstructed data. Random forest was found to be the best model with high sensitivity (100%) and specificity (99.2%). Using the Peaks-Random Forest model, we constructed an index (OCRI2) as a quantitative measurement of cancer risk. Among 11 leukoplakia patients with an OCRI2 over 0.5, 4 (36.4%) developed cancer during follow-up (23 ± 20 months), whereas 3 (5.3%) of 57 leukoplakia patients with an OCRI2 less than 0.5 developed cancer (32 ± 31 months). OCRI2 is better than other methods in predicting oral squamous cell carcinoma during follow-up. In conclusion, we have developed an exfoliative cytology-based method for quantitative prediction of cancer risk in patients with oral leukoplakia.

## INTRODUCTION

Oral squamous cell carcinoma (OSCC) is the most common histological type of oral cancer [1]. OSCC always develops from precancerous lesions such as oral leukoplakia (OLK) and erythroplakia [2]. OLK is defined as “a white plaque of questionable risk having excluded other known diseases or disorders that carry no increased risk for cancer” [3]. The overall chance of malignant transformation of OLK varies from 3.6% to 12.9% [4–6]. In contrast to a 5-year survival rate of ∼20% for advanced OSCC, the 5-year survival rate was up to 80% for OSCC diagnosed in the early stage [7]. Thus it is important to assess and follow up OLK lesions in order to diagnose OSCC early.

Several measures are available for assessment of oral cancer risk in OLK lesions. It is known that OLK lesions with ulceration or certain topography are more likely to undergo malignant transformation [8]. However, visual inspection is not reliable due to variations of physicians’ clinical experience. Histopathology (i.e., dysplasia) remains the golden standard for reporting cancer risk of OLK [9]. Unfortunately, this invasive approach depends on incisional biopsy and cannot be repeated during follow-up due to poor patient acceptance. Several other tools are also used to assess OLK lesions [10]: visual assessment of the physicochemical properties (e.g., toluidine blue staining, fluorescence spectroscopy) which is easy to use but less specific [11–13]; laboratory assessment of cellular markers (e.g., exfoliative cytology, micronucleus analysis) with higher sensitivity and specificity [14–17]; laboratory assessment of molecular markers (i.e., immunohistochemistry, gene microarray) which requires high-quality biopsy samples and are often quite expensive [18].

Exfoliative cytology is a non-invasive, easy, fast and low-cost examination for initial screening and early diagnosis of OSCC, with high sensitivity and specificity [19]. However, exfoliative cytology currently only provides qualitative assessment, instead of quantitative assessment, of cancer risk in OLK patients. In our previous study, we developed a statistical model and oral cancer risk index (OCRI) for quantitative risk of stratification of OLK patients [10]. At the time of sampling, we expected OCRI may inform us of OSCC which may be further validated by histopathology of incisional biopsy. OCRI is also expected to separate low-risk OLK from high-risk OLK, which may be followed up more frequently and in a more invasive manner, including treatment with chemopreventive agents, than low-risk OLK. Unfortunately, in our previous study on OCRI, false negative cases (i.e., 2 cases of OSCC with low OCRI values) seriously questioned the usefulness of OCRI.

In this study, by revising the method of data transformation and our preexisting statistical model, we improved the performance of risk index and eliminated false negatives. Using cytology data and clinical follow-up data of two cohorts (training set and validation set), we demonstrated that the new risk index, OCRI2, predicted OSCC much better than OCRI and the traditional method.

## RESULTS

### Peaks-Random Forest (RF) model is better than the other statistical models in differentiating normal from OSCC

**Figure 1:**
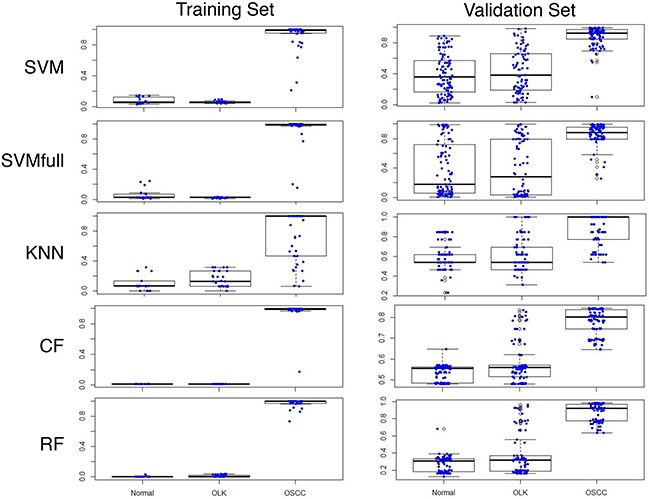
Oral cancer risk index 2 (OCRI2) of normal subjects, OLK and OSCC patients in the training and validation sets using five statistical models (SVM, SVMfull, KNN, CF and RF) Y-axis represents the value of OCRI2. Each boxplot showed the median and 25%-75% of values

After the data is transformed through peak identification, five statistical models were tested using the data of training set and validation set. As shown in Figure 1, all five models predicted the 18 normal samples correctly in the training set. Only the peaks-RF model predicted all 41 cases of OSCC correctly. For the validation set, the peaks-RF model correctly predicted 101/102 normal and all OSCC, similar to the peaks-closed forest (CF) model. The other four models had many false positives and false negatives. Since a small number of false positives can be tolerated for a cancer risk prediction model, the peaks-RF model was chosen as the statistical model for calculation of OCRI2.

### Cross-examination using the training set and the validation set confirmed the Peaks-RF model as a good prediction model of normal and OSCC

To test the validity of the peaks-RF model, we first used the training set to train our model and then tested the validation set. The peaks-RF model was able to successfully identify all 93 OSCC cases as OSCC, while only missed 1 out of 102 normal cases (sensitivity=100%, specificity=99.02% and AUC (area under the curve)=1.00). The testing process was repeated in reverse order with the validation set used for model training and the training set for prediction. The sensitivity and specificity were both 100%, while the AUC was 1.00 (Supplementary Table 1).

### Quantitative risk stratification of OLK patients and comparison of three methods

We then focused on quantitative risk stratification of OLK patients. In the training set, all 28 OLK cases were identified by the peaks-RF model as “low-risk (OCRI2<0.5)”. In the validation set, the peaks-RF model identified 64 OLKs as “low-risk (OCRI2<0.5)” and 18 OLKs as “high-risk (OCRI2≥0.5)” (Supplementary Table 2).

**Figure 2:**
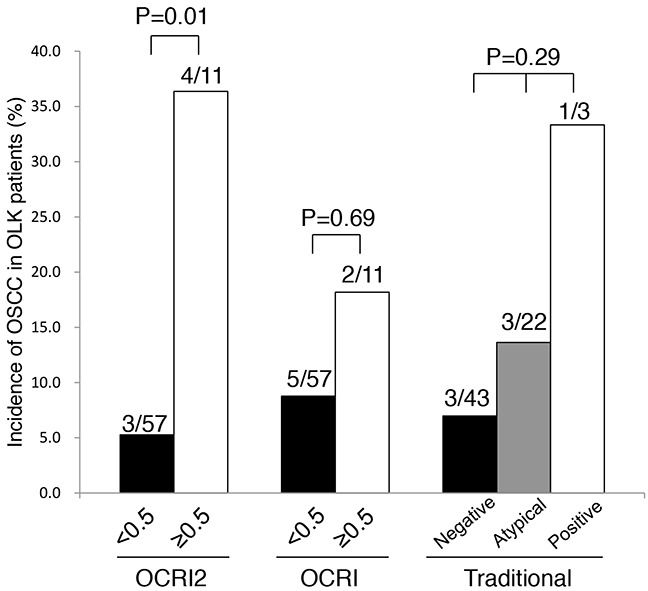
Prediction of OSCC in OLK patients during the follow-up period using three methods Seven patients developed OSCC during follow-up. With 0.5 as an arbitrary cut-off, OCRI2 predicted OSCC better than OCRI and traditional method.

We followed OLK patients in both the training set and the validation set. Among 68 cases of OLK (Supplementary Table 2), seven cases were found to undergo malignant transformation. With 0.5 as an arbitrary cut-off value for high or low risk, 36.4% (4/11) of high-risk OLK patients developed OSCC during follow-up (23±20 months), whereas 5.3% (3/57) of low-risk OLK patients developed OSCC (32±31 months) (p=0.01) (Figure 2).

We then compared the performance of OCRI2 in predicting OSCC with that of the traditional method and OCRI. Using 0.5 as an arbitrary cut-off value, OCRI failed to detect a significant difference in cancer incidence between the high-risk OLK and the low-risk OLK (p=0.69). The traditional method categorizes OLK into negative, atypical and positive groups, whose cancer risk did not significantly differ among these groups (p=0.29)(Figure 2). These data indicated that OCRI2 can predict the occurrence of OSCC in the future better than the traditional method and OCRI.

## DISCUSSION

In this study, we developed OCRI2 for assessment of cancer risk in OLK patients and had its predictive performance validated in new patients. Using two cohorts with clinical follow-up data, we demonstrated that OCRI2 can differentiate between low-risk and high-risk OLK, and may improve the cost-effectiveness during clinical follow-up of OLK patients.

Visual examination by clinicians with the aid of tools tends to have a high rate of false positivity. As a minimally invasive and inexpensive method, exfoliative cytology has long been used for qualitative detection of cervical cancer and oral cancer [20]. This approach has advantages over other methods mainly because cellular morphology tends to be relatively stable compared to molecular markers [10]. It has been well established that DNA aneuploidy could predict malignancy prior to histopathology [21, 22]. A quantitative parameter for risk stratification of OLK is desirable for two reasons: (1) Dynamic change of a quantitative parameter during clinical follow-up may add additional value towards prediction. This has been proven to be true in the case of prostate-specific antigen kinetics or velocity for prostate cancer prediction [23, 24]. (2) A quantitative parameter may improve the cost-effectiveness of clinical follow-up by paying more attention to high-risk OLK and less to low-risk OLK.

Several approaches have been used for quantitative stratification of cancer risk. Cancer risk index based on clinical risk factors using nomogram has been developed for several cancers, including head and neck squamous cell carcinoma [25], gastric cancer [26], and breast cancer [27]. In head and neck cancer, nomograms predicting five- and eight-year overall survival and cancer-specific survival were quite accurate [25]. Recently there has been tremendous enthusiasm in using molecular markers for cancer risk stratification. However, the performance of molecular markers is not much better than established clinical risk factors [28–30]. In one oral cancer study [31], a gene predictive model showed marked improvements in terms of prediction accuracy over the models using clinicopathological risk factors. Although this approach is promising, the high expense and need for special analytical expertise are obvious hurdles that must be overcome before it can be clinically useful. It is also challenging to develop a consensus gene list, according to the distinct gene lists generated by multiple studies [32].

Our study has two major limitations: (1) Three cases of OLK with OCRI2<0.5 developed OSCC. Large or multiple OLK lesions, inadequate or inappropriate sampling of exfoliative cells, mishandling of the staining and imaging procedure may all contribute to false negative prediction. Longitudinal tests of exfoliative cytology during follow-up may capture such high-risk cases, and kinetics data may have additional value in predicting cancer. Moreover, our risk index may be further improved by including multiple cytology parameters, such as nuclear perimeter, area, diameter, minimum and maximum Feret, which had been shown to be statistically different between aneuploidy and diploid samples [33]. NextGen sequencing data may also be incorporated to improve the performance of the quantitative prediction model [34, 35]. (2) Forty-two OLK patients (38%, 42/110) were lost during follow-up, among which 3 presumably were of high-risk and 39 of low-risk. This may impact the difference of true cancer risks between these two groups.

## MATERIALS AND METHODS

### Clinical information and follow-up

We recruited and followed two cohorts, the training set and the validation set, from March 2008 to July 2016. This study was approved by the ethical committee of Beijing Stomatological Hospital, Capital Medical University, and all patients signed the informed consent form before the study. Clinical data, exfoliative cytology, histopathology, and follow-up data were collected (Table 1 and Supplementary Table 2). Both exfoliative cells and incisional biopsy were collected from the site of lesion (OLK or OSCC); exfoliative cells were randomly collected from the mucosa of normal subjects. The training set was made up of 18 normal, 28 OLK and 41 OSCC subjects, and the validation set was made up of 102 normal, 82 OLK and 93 OSCC subjects (Table 1). During the follow-up, clinical symptoms of all subjects were documented through clinical examination and phones calls. Malignant transformation was confirmed by histopathology.

**Table 1:** General characteristics of subjects of the training set and the validation set

General characteristics	Training set			Validation set		
	Normal	OLK	OSCC	Normal	OLK	OSCC
Age (y)						
Mean ± SD	39.67 ± 15.48	57.68 ± 13.51	64.68 ± 11.71	44.00 ± 16.00	58.16 ± 11.48	61.70 ± 11.11
Range	20 – 68	26 - 77	32 - 88	22 - 80	25 - 85	21 - 83
Sex						
Male, n (%)	5 (27.8)	19 (67.9)	20 (48.8)	46 (45.1)	37 (45.1)	45 (48.4)
Female, n (%)	13 (72.2)	9 (32.1)	21 (51.2)	56 (54.9)	45 (54.9)	48 (51.6)
Site						
Tongue, n (%)	4 (22.2)	6 (21.4)	16 (39.0)	28 (27.5)	22 (26.8)	41 (44.1)
Gingival, n (%)	4 (22.2)	11 (39.3)	7 (17.1)	15 (14.7)	33 (40.2)	27 (29.0)
Other, n (%)	10 (55.6)	11 (39.3)	18 (43.9)	59 (57,8)	27 (32.9)	25 (26.9)
Smoking						
Yes, n (%)	1 (5.6)	16 (57.1)	10 (24.4)	32 (31.4)	29 (35.4)	31 (33.3)
No, n (%)	17 (94.4)	12 (42.9)	31 (75.6)	70 (68.6)	53 (64.6)	62 (66.7)
Drinking						
Yes, n (%)	1 (5.6)	9 (32.1)	9 (22.0)	29 (28.4)	19 (23.2)	29 (31.2)
No, n (%)	17 (94.4)	19 (67.9)	32 (78.0)	73 (71.6)	63 (76.8)	64 (68.8)
Total, n (%)	18 (100.0)	28 (100.0)	41 (100.0)	102 (100.0)	82 (100.0)	93 (100.0)

### Exfoliative cytology and histopathology

The exfoliative cytology and histopathology were conducted in the same way as in our previous study [10]. In brief, exfoliative cells were collected by using Cervibrush (Motic, China) and stored in a fixative (Motic, China). Cells were smeared onto a dry glass slide and treated with the Feulgen solution according to the manufacturer's instruction. DNA-image cytometry and CLASSIFY software (Motic, China) were used for obtained the DNA index (DI). According to the diagnostic criteria by the British Columbia Cancer Agency, an aneuploid cell was defined as DI≥2.3 [36]. A case was defined as “positive” if there were more than 5 aneuploid cells, “Atypical” if the number of aneuploid cells was between 1 and 5, or “negative” if there was no aneuploid cell. This method was defined here as the “traditional method”.

For OLK and OSCC, an incisional biopsy was taken from the same area under local anesthesia after brush biopsy. Tissues were fixed with buffered formalin and the sections were diagnosed by our pathologist according to the standard criteria of the WHO Classification System of Head and Neck Tumors [36]. Mild, moderate, or severe dysplasia of OLK is defined if the general architectural disturbance is limited to the lower third of the epithelium, extending into the middle third of the epithelium, or greater than two-thirds of epithelium, respectively [37].

### Peak determination, interval creation, and data reconstruction

Using the DI values from the sample, we first acquired the density of the data set. By taking the lagged differences of the density and observing the signs of differences, the first derivative of the DI values was mimicked. The points at which the density plot stopped rising and started falling were the positive values of the first derivative, which were then designated as peaks. These points had their x-values recorded and were compiled into a vector.

After these points were recorded, the data was reconstructed. Ten intervals were used to store the points, using a tally system that counted the number of points in each interval and returned an integer vector of 10 units long, consisting of the counts of points per interval. The intervals were created depending on the biology of cell ploidy. The intervals were divided by 10, from [0.5, 1.5], [1.5, 2.5], … to [9.5, 10.5], with any values smaller than 0.5 being included in the 1^st^ interval and any values greater than 10.5 being included in the last interval. These intervals were uniform around the natural counting numbers, 1, 2 to 10. Haploids, diploids, tetraploids and aneuploids would be easily identified with this method.

**Figure 3:**
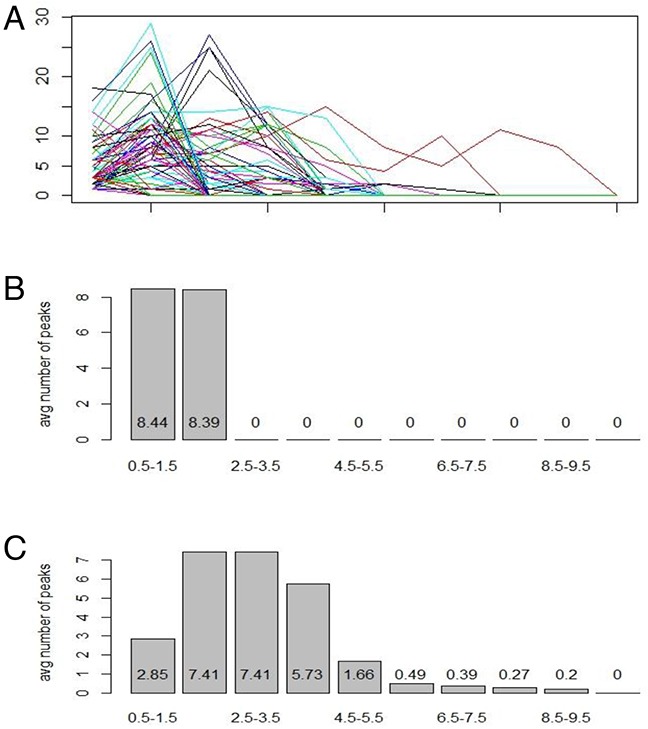
Peaks method of data transformation Peaks method was developed from first derivatives with positives defined as peaks. Ten intervals were based on ploidy value from 0.5 to 10.5, with 0.5 to 1.5 as the first interval, 1.5 to 2.5 as the second, and so on. All data of the training set (87 cases) were pooled together to show the distribution of the data **(A)**. Normal subjects (18 cases) showed two peaks in the first and second intervals **(B)**. OSCC subjects (41 cases) had high variance and their data were spread out through most of the intervals **(C)**.

After the intervals were created and the points were tallied, we had an integer vector of 10 units long, with each unit representing the number of peaks in each respective interval (Figure 3). This procedure was repeated through all the cases. Each group of data was now an X by 10 data frame, with X being the number of samples in each group. Then, 2 more columns were added, the 11^th^ of which was either a “c”, “k”, or “n” factor, representing OSCC, OLK and normal, respectively, and the 12^th^ of which was all of the case numbers of the respective samples. Thus, nX12 data frames for the training set and validation set were generated.

### Statistical models and cross examination

Statistical modeling and performance evaluation were done with R [10] and the caret package (http://caret.r-forge.r-project.org/). For the training set testing, the validation set (Normal and OSCC) were used to train the model. Firstly, the two data frames of validation set (Normal and OSCC) were combined, and then 70% of it was randomly selected for model optimization, with the remaining 30% left for testing and evaluation (with the one difference being support vector machine-full (SVMfull), which used 100% of the data). The models selected were support vector machine (SVM), SVMfull, k-nearest neighbors (KNN), CF, and RF. Along with the default parameters of the models, 10-fold cross validation was used within each pass, and the process was repeated 5 times. The same seed was also set for each random number generation, ensuring replicable results. The sensitivity and specificity of the predictions were the bases for determining which of the models was the strongest, and this was displayed in the confusion matrix. For the calculations of model adequacy, positive predictive value, negative predictive value and area under the curve were also calculated. PPV was defined as the number of cases detected as cancer that were actually cancer is divided by the total number of cases detected as cancer. For NPV, the number of cases detected as normal that were actually normal is divided by the total number of cases detected as normal. These values were found through the confusion matrix command in R. In calculating AUC, the ROCR package in R was used. Since the testing data is already ordered by factor level, the OCRIs were extracted and relabeled with their original classification. Then, the performance function was used to calculate AUC, which is between 0 and 1.

Next, the process was done in reverse. The training set (Normal and OSCC) was used to train the model, and the model was used to test the validation set (Normal and OSCC). All previous methods were implemented.

### OCRI2 calculation

OCRI2 is the probability of OSCC for an unknown sample. The range of OCRI2 ran from 0 to 1, with 0 indicating zero risk of OSCC and 1 indicating a 100% chance of OSCC. We set 0.5 as an arbitrary cut-off value to separate high-risk and low-risk cases for the sake of comparison. Two-sided Chi-square test with Yates correction was used for statistical comparison between high-risk OLK and low-risk OLK, and among “negative”, “atypical” and “positive” OLK, in Figure 2.

## SUPPLEMENTARY MATERIALS FIGURES AND TABLES




