# Supplementary material for: Quantitative prediction of oral cancer risk in patients with oral leukoplakia
Source: Oncotarget. 2017 May 2;8(28):46057–64. doi: 10.18632/oncotarget.17550 (PMC5542248; doi:10.18632/oncotarget.17550)
Supplement: Supplementary file 1 [file oncotarget-08-46057-s001.pdf]

Quantitative prediction of oral cancer risk in patients with oral leukoplakia

SUPPLEMENTARY TABLES

Supplementary Table 1: Sensitivity, specificity, PPV, NPV and AUC of cross-examining the training set and the validation set

Modeling → Calculation [a]	Sensitivity (%)[b]	Specificity (%)[c]	PPV (%)[d]	NPV (%)[e]	AUC[f]
Training set → Validation set	100.00	99.02	98.94	100.00	1.00
Validation set → Training set	100.00	100.00	100.00	100.00	1.00

[a] "Training set → Validation set" refers to modeling with the training set and calculating OCRI2 with the validation set. "Validation set → Training set" refers to modeling with the validation set and calculating OCRI2 with the training set
[b] Sensitivity is calculated as the number of OSCC cases with an OCRI2 ≥ 0.5 divided by the number of OSCC cases.
[c] Specificity is calculated as the number of normal cases with an OCRI2 < 0.5 divided by the number of normal cases.
[d] PPV (Positive predictive value) is defined as cancer being predicted as cancer.
[e] NPV (Negative predictive value) is defined as normal being predicted as normal.
[f] AUC: Area Under the Curve.

Supplementary Table 2: Raw data of the training set and the validation set (Excel spreadsheets).

See Supplementary File 1
